# Peer Review of “Monte Carlo Dose Estimation of Absorbed Dose to the Hematopoietic Stem Cell Layer of the Bone Marrow Assuming Nonuniform Distribution Around the Vascular Endothelium of the Bone Marrow: Simulation and Analysis Study”

**DOI:** 10.2196/77775

**Published:** 2025-07-16

**Authors:** Randa Salah Gomaa Mahmoud

**Affiliations:** 1 Zagazig University, Zagazig, Egypt

**Keywords:** stem cells, radiation, bone marrow, nuclides, noble gases

*This is the peer-review report for “Monte Carlo Dose Estimation of Absorbed Dose to the Hematopoietic Stem Cell Layer of the Bone Marrow Assuming Nonuniform Distribution Around the Vascular Endothelium of the Bone Marrow: Simulation and Analysis Study.”*

## Round 1 Review

### General Comments

In this study [1], a geometric model of trabecular bone and bone marrow tissue was constructed at the micrometer scale, assuming that the hematopoietic stem cells layer was localized in the perivascular hematopoietic stem cell layer of the sinusoids. The absorbed doses of the stem cell layer from blood and trabecular bone sources were then estimated for selected β nuclides, α nuclides, and noble gases and compared with the specific absorbed fractions (SAFs) values of International Commission on Radiological Protection (ICRP) 60 and 103. It was concluded that the absorbed doses from the bone marrow and blood sources were greater than those from trabecular bone sources for α nuclides, and the total absorbed dose was lower than that estimated from the current ICRP models.

### Specific Comments

1. The results were tabulated; however, it was not clear how the comparison between the Particle and Heavy Ion Transport System, ICRP 60, and ICRP 103 was performed, what test was used, and the level of significance. Even in Table 7 that summarizes the results, this is not clear.
2. The abbreviations throughout the article need to be identified. It is recommended to add an abbreviation section to the article.
3. The abstract section is better structured as Background, Objectives, Methods, Results, and Conclusion.
4. In the abstract section, the authors mentioned that the absorbed doses to the bone marrow obtained from the model calculations were not significantly different from ICRP 60 and ICRP 103 for β nuclides. Still, they were much lower than previously estimated for α nuclides. Going through the study, it was not clear how this significant difference was assessed. Please revise and clarify.
5. The abbreviation “SAFs” in the keyword section and the last paragraph of the Introduction section should be identified as the “specific absorbed fractions.”
6. The abbreviation “PHITS” in the keyword section and the first line of the fourth page should be identified as ”Particle and Heavy Ion Transport System.”
7. The abbreviation “keV” in the last line of the second paragraph of the seventh page should be identified as “kilo electron-volt.”
8. In the last line of the second paragraph of the seventh page, please identify “Bremsstrahlung” as a type of X-radiation emitted by charged particles when they collide or are near an atomic nucleus.
9. The abbreviation “EGS” in the last line of the second paragraph of the seventh page should be identified as “Electron Gamma Shower.”
10. The abbreviation “Bq” in the first line of the last paragraph of the seventh page should be identified as “The International System of Units (SI) unit of radionuclide activity is the becquerel (Bq); 1 Bq = 1 transformation/second.”
11. First line, page 10: Please correct “131” to “131I.”
12. Page 16, Discussion section, last line of the first paragraph: The authors mentioned that the number of decays in each compartment changed significantly; how did the authors assess this significant change and conclude it? Please explain the tests used for comparison.
13. Page 16, Discussion section, eighth line of the second paragraph: Please revise “ICRP133 SAF” (mentioned in the Results section as “ICRP103 SAF”).
14. Page 17, last line of the first paragraph: “Sakota et al” should be corrected to “Sakoda et al.”

## Round 2 Review

### General Comments

All the comments were professionally addressed.

## Abbreviations

ICRP: International Commission on Radiological Protection
SAF: specific absorbed fraction
SI: International System of Units
